# Highly Efficient Spatial Three-Level CoP@ZIF-8/pNF Based on Modified Porous NF as Dual Functional Electrocatalyst for Water Splitting

**DOI:** 10.3390/nano13081386

**Published:** 2023-04-17

**Authors:** Hongzhi Wang, Limin Zhang, Weiguo Zhang, Shaofeng Sun, Suwei Yao

**Affiliations:** 1 Department of Applied Chemistry, School of Chemical Engineering and Technology, Tianjin University, Tianjin 300350, China; wanghz@tju.edu.cn (H.W.);; 2 Institute of Sport and Health, Tianjin University of Sport, Tianjin 301617, China; 3 Fortune LMS Ltd., Calgary, AB T1W 0K6, Canada

**Keywords:** spatial three-level structure, CoP@ZIF-8, porous NF, HER, OER, overall water splitting

## Abstract

The development of non-noble metal catalysts for water electrolysis to product hydrogen meets the current strategic need for carbon peaking and carbon neutrality. However, complex preparation methods, low catalytic activity and high energy consumption still limit the application of these materials. Herein, in this work we prepared a three-level structured electrocatalyst of CoP@ZIF-8 growing on modified porous nickel foam (pNF) via the natural growing and phosphating process. In contrast to the common NF, the modified NF constructs a large number of micron-sized pores carrying the nanoscaled catalytic CoP@ZIF-8 on the millimeter-sized skeleton of bare NF, which significantly increases the specific surface area and catalyst load of the material. Thanks to the unique spatial three-level porous structure, electrochemical tests showed a low overpotential of 77 mV at 10 mA cm^−2^ for HER, and 226 mV at 10 mA cm^−2^ and 331 mV at 50 mA cm^−2^ for OER. The result obtained from testing the electrode’s overall water splitting performance is also satisfactory, needing only 1.57 V at 10 mA cm^−2^. Additionally, this electrocatalyst showed great stability for more than 55 h when a 10 mA cm^−2^ constant current was applied to it. Based on the above characteristics, the present work demonstrates the promising application of this material to the electrolysis of water for the production of hydrogen and oxygen.

## 1. Introduction

As fossil energy sources become increasingly depleted, hydrogen energy, as a clean, efficient and sustainable new energy source, has received widespread attention and research from the scientific community [1,2,3]. Electrochemical water splitting is a common method of preparing hydrogen, and the product is still water after the hydrogen has been used, which is sustainable and holds great promise for research [4,5,6,7,8,9]. According to research, the theoretical decomposition voltage of water is 1.23 V. However, in the actual industrial electrolysis of water for hydrogen production, the applied voltage is much larger than 1.23 V due to the overpotential of hydrogen evolution reaction (HER) and oxygen evolution reaction (OER), which increases the energy consumption of electrolytic water for hydrogen production [10,11,12,13]. To reduce the reaction overpotential, focusing on electrode materials that are relevant and commercially viable is a key issue in solving the problem of large energy consumption [14,15,16]. At present, noble metal-based electrocatalysts are the most efficient electrocatalysts for HER and OER reactions, but the high cost and limited natural reserves have limited their large-scale application [17,18,19,20]. Hence, the development of inexpensive catalysts with high energy efficiency and stability is essential as a possible alternative to noble metals.

In recent years, phosphides of transition metals have become a hot research subject in the field of electrocatalyst for HER and OER [21,22,23,24,25]. Among these, the excellent catalytic properties exhibited by phosphorylates of cobalt and their derivatives have attracted more attention from researchers [26,27,28]. Xie et al. [29] designed a two-dimensional material CoP/CNFs based on the growth of carbon nanofibers (CNFs) for water splitting, achieving well electrocatalytic performance. Li et al. [30] prepared a CoP/C with octahedral particle structure, whose electrocatalytic activity can be maintained for more than 24 h. Due to the large radius of the phosphorus atom (0.109 nm), its crystal structure is trigonal, forming an isotropic crystal structure rather than a layered one, resulting in a number of coordination-unsaturated surface atoms [31]. In addition, because of the low electronegativity of phosphorus, cobalt phosphide has not only covalent bonds, but also metallic bonds and even chemical bonds with semiconductor properties, which makes its electrical conductivity, activity and stability excellent [32,33]. However, the preparation methods of cobalt phosphide mostly adopt the hydrothermal method and high-temperature phosphating method, the microstructure of the product is not easy to control, and the reaction area is limited. Currently, the excellent performance is mainly reflected in one of HER and OER, and there are not enough strategies that can provide well electrocatalytic activity in these two reactions [34,35].

Metal–organic frames (MOF) are crystalline and porous materials formed by organic ligands with metal ions that prove to be an excellent template for the synthesis of compounds of various metals [36,37,38]. Among them, ZIFs are a type of zeolite imidazolate-type 3D skeleton structure material with a high content of transition metal ions and abundant pores and channels [23,39,40]. The central ion of ZIF-67 is cobalt ion, which is an ideal intermediate for the synthesis of porous CoP materials [41,42,43]. However, most of the current popular synthesis methods require more than one high-temperature reaction step (mostly above 600 °C) or other complex doping reactions, and the resulting products are mostly powdered, the reaction energy consumption is high and the process is complex, which is not conducive to large-scale industrial application [41,44,45]. ZIF-8, which has a similar structure, is often used for surface modification of various nanomaterials, which can control the nucleation size and morphology of the modified objects. Meanwhile, its high stability can enhance the overall structural stability of the product [46,47].

Porous metal materials are special materials composed of metals and pores with high porosity, high specific surface area, high electrical conductivity and high adsorption, which have a wide range of applications in the fields of batteries, electrocatalysis, sensors, etc. [48,49,50]. The dynamic hydrogen bubble template (DHBT) method is currently a popular and simple strategy used to prepare porous metal materials. It is used to construct porous structures of metals by using the hydrogen bubbles that overflow during the cathodic process of the electrodeposition reaction as a template, which has the advantages of low reaction temperature, short time and easy operation compared to common electrodeposition [50,51,52]. This method is often used to prepare porous materials of nickel, and porous nickel itself has a certain electrocatalytic performance for HER [53,54,55]. However, the preparation of porous nickel is mainly grown on flat metallic substrates and is correspondingly less explored in the deposition of nickel on uneven substrates. In addition, the current research works mainly focuses on the catalytic performance of the porous metal materials themselves, with fewer further applications using them as carriers.

Based on the above inspirations, we synthesized the modified porous nickel foam (pNF) by direct and rapid electrodeposition of nickel on NF using the hydrogen bubble template method. Then, the CoP@ZIF-8 electrocatalyst with a nanoscale core–shell structure was prepared in the micron pore of the pNF as the carrier using the natural growth method and low temperature phosphatizing method, which integrated the catalytic effect of both the carrier itself and the nano-active substance. The overpotential for HER and OER of this electrocatalyst is 77 mV at 10 mA cm^−2^ and 331 mV at 50 mA cm^−2^, indicating that it has the prospect of industrial application for hydrogen production. 

## 2. Materials and Methods

### 2.1. Chemicals

Cobalt nitrate hexahydrate (Co(NO_3_)_2_·6H_2_O, 99.9%), zinc nitrate hexahydrate (Zn(NO_3_)_2_·6H_2_O, 99.9%), 2-methylimidazole (C_4_H_6_N_2_, 99.9%), nickel(II) chloride (NiCl_2_, 99%), sodium chloride (NaCl, 99%), ammonium chloride (NH_4_Cl, 99%), potassium hydroxide (KOH, 99%) and sodium hypophosphite (NaH_2_PO_2_·H_2_O, 99%) were purchased from Macklin Biochemical Co., Ltd. (Shanghai, China). Sodium dodecyl sulfate (C_12_H_25_SO_4_Na, 99%), hydrochloric acid (HCl, 99%) and nickel foam were sourced from Kemate Chemical Technology Co., Ltd. (Tianjin, China). All other chemicals used in the experiment were analytical grade and purchased from commercial suppliers.

### 2.2. Preparation of CoP@ZIF-8/pNF Electrode

The CoP@ZIF-8/pNF electrode was prepared by rapid electrodeposition, natural growing and the phosphating method. First, the pNF was prepared by constant current electrodeposition. The NF (1 cm × 2 cm) was de-oiled by a KOH solution and cleaned by hydrochloric acid and deionized water. Then, a 1 A/cm^2^ current was applied for 60 s to the 0.2 M NiCl_2_, 1 M NaCl, 2 M NH_4_Cl and 0.2 C_12_H_25_SO_4_Na electrolytes (pH = 3) at room temperature. After deposition, the pNF was washed with deionized water. Second, the as-prepared the pNF was put into 60 mL 2 M 2-methylimidazole solution and stirred magnetically with a small magnet for 10 min. Then, 30 mL 0.1 M Co(NO_3_)_2_ was poured into the above solution. After 2 min, 30 mL 0.1 M Zn(NO_3_)_2_ was added. After 1 h of stirring, the stirring stopped. The mixture solution then stood at room temperature for 24 h. The ZIF-67@ZIF-8/pNF obtained from the solution was cleaned with deionized water and put into a vacuum oven at 60 °C for 12 h to remove water.

**Figure 1:**
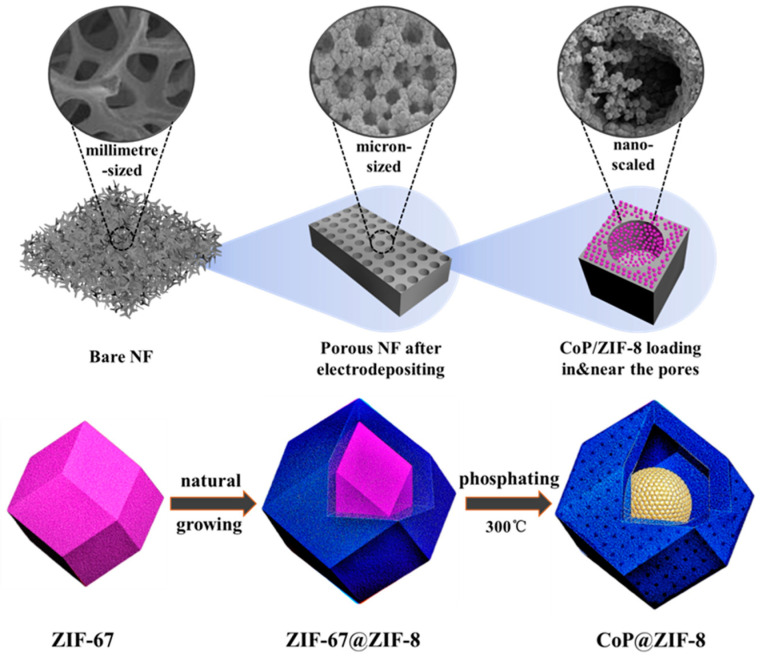
The spatial three-level structure of the CoP@ZIF-8/pNF and schematic illustration of the synthesis of the CoP@ZIF-8.

Finally, 2.0 g NaH_2_PO_2_·H_2_O and the ZIF-67@ZIF-8/pNF were placed upstream and downstream in the tube furnace, respectively. The temperature in the tube furnace was increased to 250 °C at a rate of 5 °C/min and then up to 300 °C at 1 °C/min and maintained for 2 h to phosphatize the precursor before cooling down naturally. Finally, the CoP@ZIF-8/pNF electrode was prepared. Figure 1 shows a schematic diagram of the preparation process of the CoP@ZIF-8/pNF electrode. The electrocatalyst has a mass loading of about 0.8 mg cm^−2^.

The CoP@ZIF-8/NF electrode was prepared by using the same method with a 1:1 Co-Zn mole ratio as for CoP@ZIF-8/pNF electrode, except that bare NF was used instead of the pNF. The CoP/pNF electrode was prepared by using the same method as for CoP@ZIF-8/pNF, except that Zn(NO_3_)_2_ was not added and the amount of 2-methylimidazole was halved.

### 2.3. Materials Characterization

X-ray diffraction (XRD, Cu Kα radiation, λ = 0.15406 nm) ranging from 5° to 80° (2θ) was used to examine the crystallization and compounding of the CoP@ZIF-8/pNF electrocatalyst. Additionally, it was characterized by field emission scanning electron microscopy (FESEM, Hitachi S4800, Tokyo, Japan) and a field emission transmission electron microscope (FETEM, JEOL JEM-F200, Tokyo, Japan). X-ray photoelectron spectrometry (XPS) measurements of the electrocatalyst were taken on Thermo K-Alpha+ (Thermo Scientific, Waltham, MA, USA) to identify the composition of the electrocatalyst.

Additionally, CoP@ZIF-8 was scraped off the pNF by continuous ultrasound in an anhydrous ethanol solution. The powder was then collected by centrifugation before being washed and dried. The resulting sample was used for XRD, TEM and XPS characterization. To measure the interior, the powder sample was etched by Ar plasma using a precision ion polishing system (Gatan 695, Pleasanton, CA, USA) for XPS testing.

### 2.4. Electrochemical Measurements

Using an electrochemical workstation (Chenhua CHI660E, Shanghai, China), the electrochemical measurements were taken in 1 M KOH solution. The CoP@ZIF-8/pNF electrode, saturated calomel electrode (SCE) and Pt foil served as the working electrode, reference electrode and counter electrode, respectively. Linear sweep voltammetry (LSV) with a scan rate of 2 mV s^−1^ was performed in a range from −0.6~0 to 1.2~2.0 V (vs. RHE) to measure the performance of the electrocatalyst. The electrochemical impedance spectroscopy (EIS) tests were executed with an AC voltage of 5 mV ranging from 2–10 to 105 Hz. Serving as both the anode and the cathode, the electrocatalysts in this work were tested at a voltage ranging from 1.2 to 2.0 V to obtain the performance of the overall water splitting. Additionally, the water splitting stability of the CoP@ZIF-8/pNF electrocatalyst was tested by applying a constant current density of 10 mA cm^−2^, which was achieved by the LAND System (Wuhan) to detect voltage changes. Obtaining the electrochemical double-layer capacitance (Cdl) was achieved by cyclic voltammetry (CV) measurements with different scan rates (10, 20, 30, 40, 50, 60 and 70 mV s^−1^) in the non-Faradaic region (0.1–0.2 V vs. RHE) [56]. CV measurements were also used to test the stability of the electrocatalysts under changing voltages of −0.6~0 V and 1.2~2.0 V (vs. RHE) at a scan rate of 10 mV s^−1^ for HER and OER. All potential values were calculated via the following equation: E (vs. RHE) = E (vs. SCE) + 0.0592pH + 0.241 V.

## 3. Results

### 3.1. The Synthesis Mechanism and Characterization of the pNF

In the reaction of nickel metal electrodeposition using the DHBT method, the following reactions take place on the cathode surface [51], where (aq) denotes a substance in free aqueous solution, (ads) denotes a substance adsorbed on the surface of a solid and (s) denotes the solid substance:

(1) $$2H_{\left(aq\right)}^{+}+2e^{-}\to H_{2}$$

(2) $$H_{\left(aq\right)}^{+}+e^{-}\to H_{\left(ads\right)}$$

(3) $$H_{\left(aq\right)}^{+}+H_{\left(ads\right)}+e^{-}\to H_{2}$$

(4) $$2H_{\left(ads\right)}\to H_{2}$$

(5) $${Ni}_{\left(aq\right)}^{2+}+2e^{-}\to {Ni}_{\left(s\right)}$$

Formulas (1)–(4) explains the mechanism by which H^+^ is converted into H_2_. Formula (5) is the process of nickel ion deposition on the substrate through the electron reaction. On the surface of the solid nickel metal, the reaction to produce deposits of nickel and the reaction to produce hydrogen take place simultaneously, disturbing the growth of nickel. The bubbles resting on the surface of the solid accumulate from small to large until the decreasing contact angle is sufficiently small to detach and act as a template for the pores. The morphology and properties of the products obtained from the preparation of porous NF by the DHBT method was mainly influenced by the current density and deposition time, in addition to the electrolyte concentration, additives, etc.

**Figure 2:**
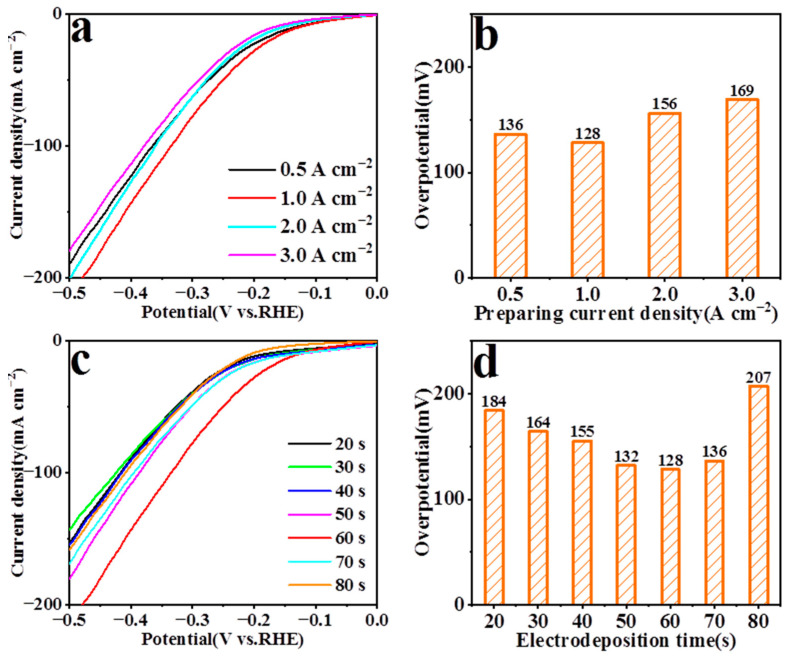
The LSV curves (**a**) and the overpotential at 10 mA cm^−2^ (**b**) for HER of the pNF prepared at different current densities for 60 s. The LSV curves (**c**) and the overpotential at 10 mA cm^−2^ (**d**) for HER of the pNF prepared for different electrodeposition times under 1.0 A/cm^−2^.

**Figure 3:**
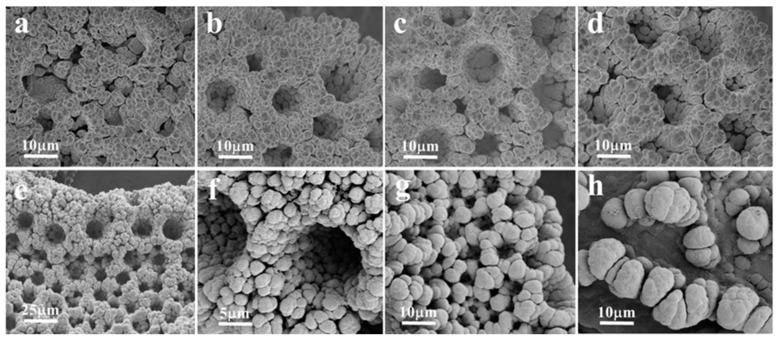
SEM images of the pNF prepared at 1.0 A cm^−2^ for different electrodeposition times. (**a**) 20 s, (**b**) 30 s, (**c**) 40 s, (**d**) 50 s, (**e**,**f**) 60 s, (**g**) 70 s, (**h**) 80 s.

In order to obtain a electrocatalyst with better performance using the pNF as the substrate, linear sweep voltammetry (LSV) and SEM were employed to explore the HER performance of the pNF at different preparing current densities and different electrodeposition times. The electrocatalytic performance of the pNF for HER is shown in Figure 2. When the preparing current density was 0.5 A/cm^−2^, the lower precipitation potential of H^+^ led to less hydrogen generated, and the small amount of bubbles on the NF surface was not enough to generate a 3D porous structure [51]. As the current density gradually increased up to1.0 A/cm^−2^ or more, enough hydrogen was generated to be used as a template to generate stable pores (Figure 3). Among them, the porous structure of the pNF prepared at 1.0 A/cm^−2^ had the best HER performance, which had an overpotential of 128 mV at 10 mA cm^−2^ (Figure 2a,b). As shown in Figure 2c,d, the optimum electrodeposition time at 1.0 A/cm^−2^ for the preparation of the pNF was 60 s. 

SEM images of the pNF prepared at 1.0 A cm^−2^ for different electrodeposition times are shown in Figure 3. When the electrodeposition time was too short (Figure 3a–d), the stable pores structure had not yet been formed and a small number of concave holes formed on the surface of the deposit layer. On the contrary, when the electrodeposition time was too long, the hydrogen bubbles showed a sharp increase and occupied the position of the original nickel grains, which could cause the distance between the nickel grains to be too wide, causing the porous structure to collapse (Figure 3g,h). As shown in Figure 3e,f, the pore structure of the pNF was obvious, and dense and uniform pores of 5–10 microns in size were formed on the surface of the nickel foam after 60 s of electrodeposition. The common NF was modified and the pNF with micron pores was prepared successfully.

### 3.2. The Synthesis Mechanism of CoP@ZIF-8

Figure 1 also shows a schematic illustration of the CoP@ZIF-8. In aqueous solution, cobalt nitrate (Co(NO_3_)_2_) and 2-methylimidazole (C_4_H_6_N_2_) formed ZIF-67, which occupied the inner site of the core–shell structure as the precursor of CoP. ZIF-8, formed from zinc nitrate (Zn(NO_3_)_2_) and excess 2-methylimidazole (C_4_H_6_N_2_) in the solution, was wrapped around ZIF-67 as the outer shell by natural growing. The same organic ligand (2-methylimidazole) and basically the same crystal structure allowed ZIF-8 and ZIF-67 to form a stable atom-compatible core–shell heterostructure [57]. Due to the different values of reduction energy of Zn (E_reduction_ = −0.5446 eV) and Co (E_reduction_ = 1.0036 eV), PH_3_ produced by the heating decomposition of NaH_2_PO_2_·H_2_O in the tube furnace could selectively reduce ZIF-67 while not reacting with ZIF-8 [58]. Additionally, the abundant pore structure of the ZIFs ensured the uniform diffusion of PH_3_ in ZIF-67@ZIF-8. While the internal CoP was obtained, ZIF-8 retained its overall structure and its Zn-N bonds were partially broken when calcined at 300 °C [59], which allowed more pores and channels to grow on the surface of ZIF-8. ZIF-8 was often used as the modification component of HER and OER electrocatalysts due to its unique structure and abundant hydrogen adsorption sites [39]. The structure of ZIF-67 would collapse in the process of low temperature phosphating [23], so ZIF-8 could control the size and surface charge of internal CoP to prevent phosphide agglomeration. CoP was assembled by ZIF-8 into many individual electrocatalytic units, which further increased the reactive area and was conducive to bubble growth desorption. Meanwhile, the great oxidation resistance of ZIF-8 itself ensured the overall stability of the material.

### 3.3. The Effects of Different Co-Zn Ratios

**Figure 4:**
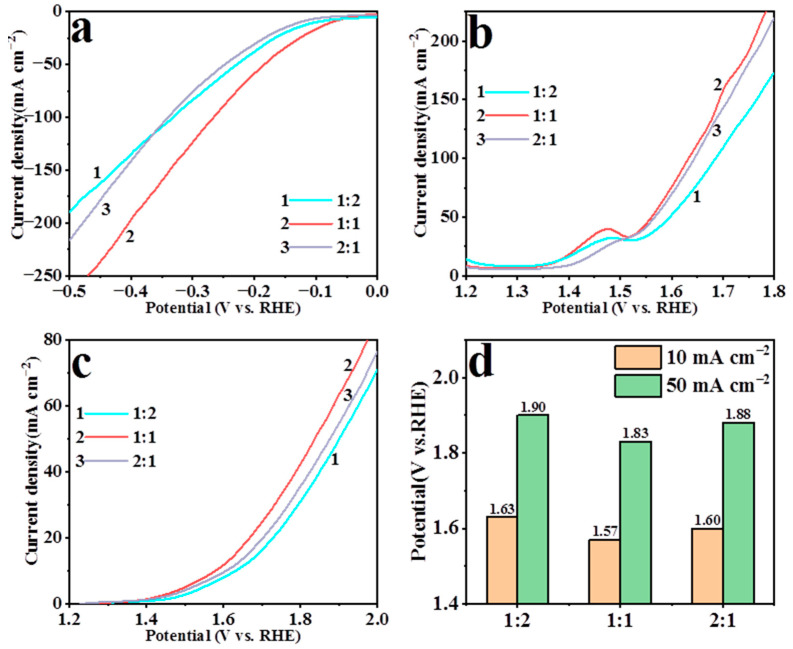
The LSV curves for HER (**a**) and OER (**b**) of CoP@ZIF-8/pNF prepared with different Co-Zn ratios. The LSV curves for water splitting (**c**,**d**) of CoP@ZIF-8/pNF (+||−) prepared with different Co-Zn ratios.

The Co-Zn mole ratios in the solution were 1:2, 1:1 and 2:1, which was controlled by the usage of cobalt nitrate and zinc nitrate. As shown in Figure 4, the prepared electrocatalysts achieved the best HER and OER performance when the ratio was 1:1. When the ratio was 1:2, excess external ZIF-8 led to an excessive shell thickness on the outside of the electrocatalyst, preventing sufficient contact between the CoP and the electrolyte. Conversely, too much CoP would agglomerate in the interior and reduced its catalytic activity and conductivity when the ratio was 2:1.

### 3.4. Microstructure and Morphology

**Figure 5:**
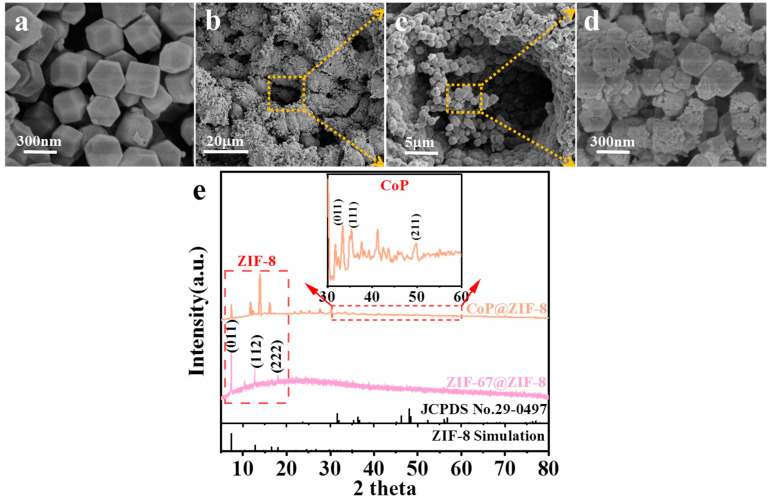
SEM images of the ZIF-67@ZIF-8 (**a**) and CoP@ZIF-8/pNF (**b**–**d**); XRD patterns (**e**) of the ZIF-67@ZIF-8 and CoP@ZIF-8.

Figure 5a shows the SEM images of nano-scaled ZIF-67@ZIF-8 presenting a regular dodecahedron structure. There were pores on the NF with apertures ranging from 10 to 20 μm, and CoP/ZIF-8 grew in and near the pores (Figure 5b). As shown in Figure 5c, CoP@ZIF-8 successfully grew in and near the micron-sized pores on the pNF without blocking the internal channel of the pores. After phosphating at 300 °C, the partial fracture of the Zn-N bond in ZIF-8 caused slight irregularities in its morphology, and the increase of surface pores aggravated the changes (Figure 5d). Therefore, CoP@ZIF-8/pNF had a large reactive area and reaction site, and integrated the catalytic activity of both the pNF and CoP@ZIF-8. The pNF also acted as a fluid collector. The corresponding XRD patterns of ZIF-67@ZIF-8 and CoP@ZIF-8 are shown in Figure 5e. Because the pNF had a strong background, ZIF-67@ZIF-8 and CoP@ZIF-8 were scraped off from the pNF. The diffraction peaks located at 7.4°, 12.8° and 18.1° are well-assigned to the (011), (112) and (222) planes of ZIF-8, respectively. Because the signal of ZIF-8 is too large, the detected diffraction peaks of CoP are relatively weak. In the enlarged figure, three diffraction peaks of 31.6°, 36.3° and 48.1° can be well-indexed to the (011), (111) and (211) crystal planes of CoP (JCPDS No.29-0497), respectively.

**Figure 6:**
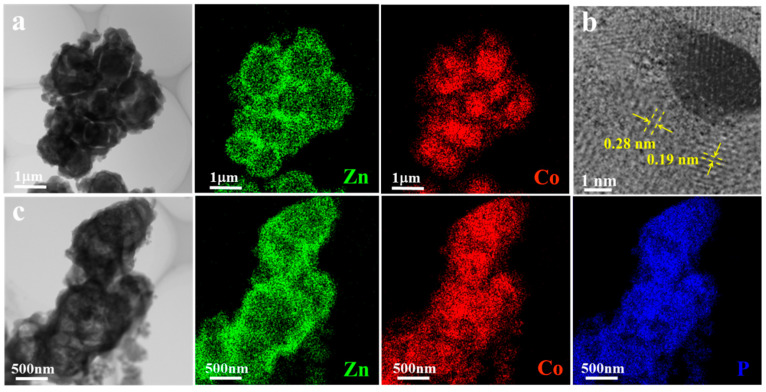
(**a**) TEM-EDS mapping of the ZIF-67@ZIF-8 of Zn, Co. (**b**) HRTEM image of the CoP@ZIF-8. (**c**) TEM-EDS mapping of the CoP@ZIF-8 of Zn, Co, P.

Corresponding EDS mapping results (Figure 6a) of the low-TEM image show that the shell of ZIF-67@ZIF-8 is mainly composed of Zn elements, which means that ZIF-8 wraps perfectly around the internal ZIF-67. Additionally, the HRTEM image of the CoP@ZIF-8 scratched off from the pNF is shown in Figure 6b. It shows that the distances of the lattice fringes are 0.19 and 0.28 nm, corresponding to the (211) and (011) crystal planes of CoP. Similarly, in Figure 6c, the elemental distributions of P and Co overlap, with CoP being in the inner part of the core–shell structure. By contrast, the distribution of the element Zn still indicates that ZIF-8 is distributed in the outer layer of the material. These results also demonstrate the successful preparation of the core–shell structure of CoP@ZIF-8 on the pNF.

**Figure 7:**
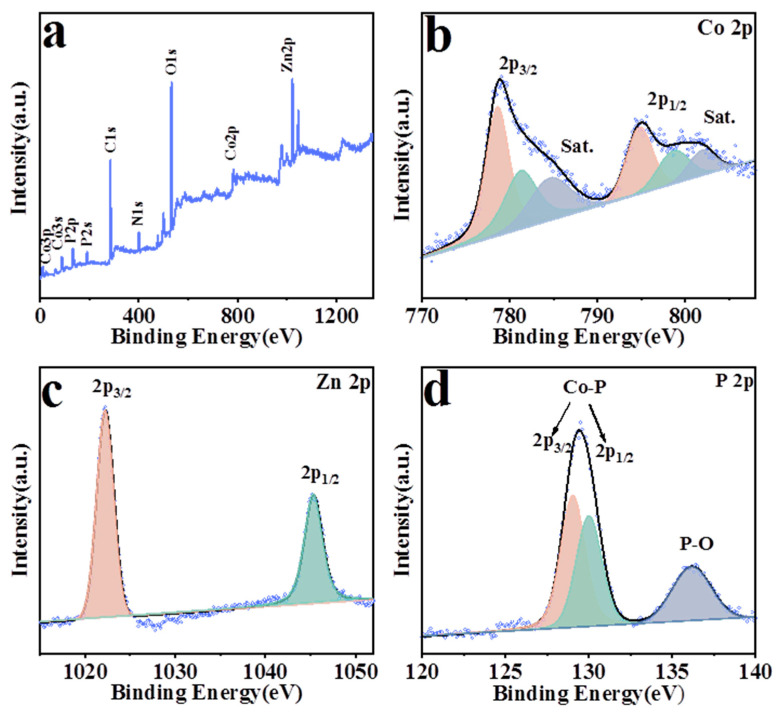
XPS spectra of CoP@ZIF-8. (**a**) Full scan XPS survey; high-resolution XPS signals of (**b**) Co 2p; (**c**) Zn 2p; (**d**) P 2p.

X-ray photoelectron spectrometry (XPS) tests were used to perform component analysis and electronic states’ characterization of CoP@ZIF-8. As shown in Figure 7a, the survey spectrum of CoP@ZIF-8 proved the existence of Co, Zn and P elements. Figure 7b shows that the binding energies of the Co 2p_3/2_ peaks located at 778.8, 781.8 and 786.4 eV could be assigned to the Co banded to P in CoP, oxidized Co species and the satellite peak, respectively. In the Co 2p_1/2_ part, the peaks of the synthesized CoP@ZIF-8 had peak assignments similar to those of Co 2p_3/2_, whose positions of peaks at 794.2, 799.2 and 803.4 eV could be attributed to Co-P, oxidized Co species and satellite peak, respectively [60]. Two peak positions at 1022.1 eV (Zn 2p_3/2_) and 1045.1 eV (Zn 2p_1/2_) can be seen in Figure 7c, corresponding to the Zn^2+^ in ZIF-8 [59]. In Figure 7d, three peaks of P 2p are located at 129.2, 129.8 and 135.8 eV. Due to the P with a partial negative charge, the binding energies of P 2p_3/2_ and P 2p_1/2_ were shifted negatively [61]. The peak shifts illustrate the generation of CoP and the shifting of electron clouds, which facilitated the capture of more protons during the electrocatalytic process. Additionally, the P-O peak could be ascribed to the oxidation on the surface of CoP@ZIF-8.

### 3.5. Hydrogen Evolution Reaction

**Figure 8:**
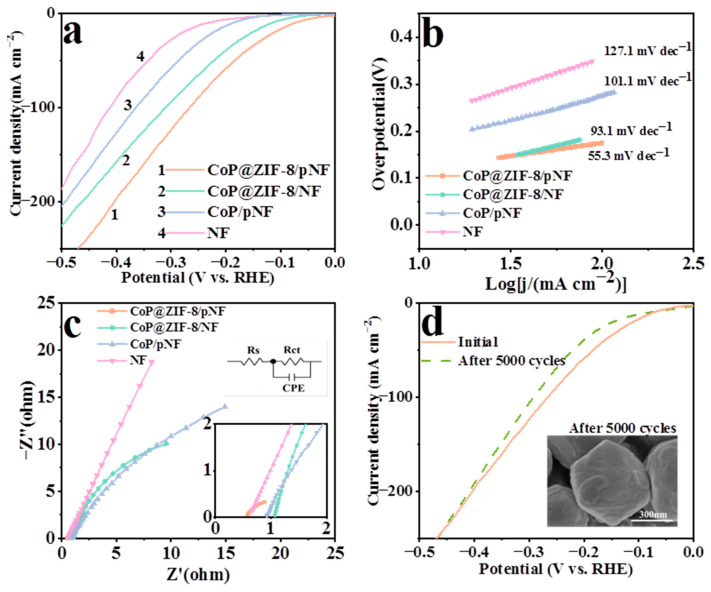
LSV curves (**a**), the corresponding Tafel plots (**b**) and EIS test (**c**) for HER of CoP@ZIF-8/pNF, CoP@ZIF-8/NF, CoP/pNF and bare NF in 1.0 M KOH. (**d**) LSV for HER of CoP@ZIF-8/pNF after 5000 CV cycles.

As shown in Figure 8, the cathodic HER process was evaluated in a three-electrode system in 1M KOH by using a CHI660 workstation at room temperature. In Figure 8a, the curve of CoP@ZIF-8/pNF shows the best HER performance with 77 mV at 10 mA cm^−2^, while CoP@ZIF-8/NF presents a modest performance with an overpotential of 116 mV at 10 mA cm^−2^. Additionally, the overpotentials of 187 and 234 mV means CoP/pNF and bare NF have poor electrocatalytic activity. The excellent performance of CoP@ZIF-8/pNF indicates that CoP can fully contact and react with the electrolyte in the core–shell structure covered with pores, and the abundant pore structure is conducive to the rapid desorption of the generated hydrogen from the catalyst surface. Since NF does not have as many micron-sized pores as the pNF, there is not an adequate area on the NF where the catalyst can be effectively loaded, resulting in limited performance of CoP@ZIF-8/NF. The Tafel plots and EIS test also support the existence of these reasons. As can be seen in Figure 8b, the low Tafel slope of 55.3 mV dec^−1^ of CoP@ZIF-8/pNF also demonstrates its faster reaction kinetics and high catalytic activity, obeying the Volmer–Heyrovsky mechanism [62]. The other three Tafel slopes are 93.1, 101.1 and 127.1 mV dec^−1^, corresponding to their moderate catalytic activity. In the Nyquist plots, R_CT_ presents the charge transfer resistance, which is related to the catalytic kinetics, and R_S_ indicates the solution resistance, which is one of the reasons for the increased voltage drop of the reaction. In Figure 8c, the R_CT_ of CoP@ZIF-8/pNF is 1.16 Ω, which is far lower than the other three catalysts. The low R_CT_ suggests that the special structure of CoP@ZIF-8/pNF facilitates efficient charge transfer. In addition to the efficient electrocatalytic capability, stability is also an important factor in the suitability of the catalyst for future applications. As shown in Figure 8d, the cyclic voltammetry (CV) can be used to test catalyst stability. The LSV curve measured after 5000 cycles shows little changes compared with the initial performance, and the SEM image after cycling also shows slight structural changes, showing good chemical stability, which is related to the stable crystal lattice in CoP and the resistance of the ZIF-8 shell.

### 3.6. Oxygen Evolution Reaction

**Figure 9:**
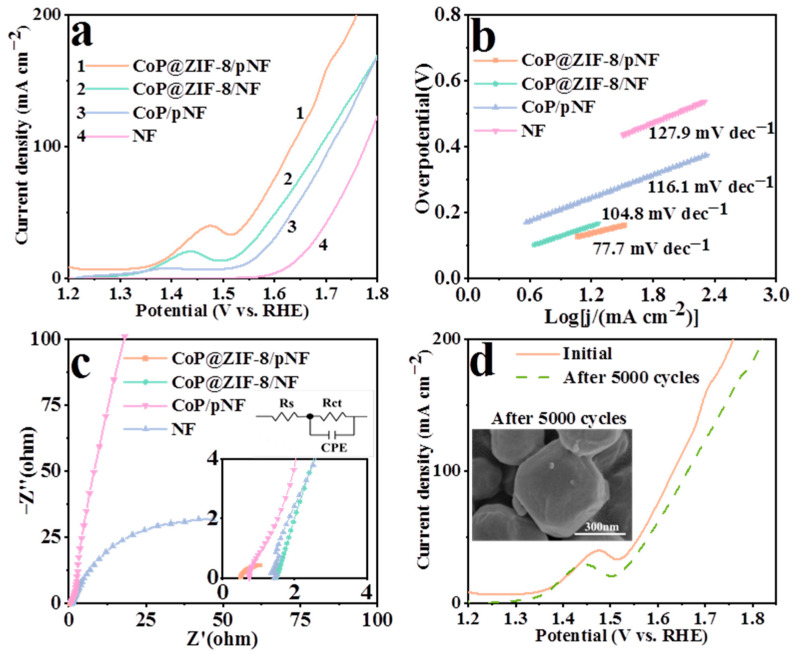
LSV curves (**a**), the corresponding Tafel plots (**b**) and EIS test (**c**) for OER of CoP@ZIF-8/pNF, CoP@ZIF-8/NF, CoP/pNF and bare NF in 1.0 M KOH. (**d**) LSV for OER of CoP@ZIF-8/pNF after 5000 CV cycles.

CoP@ZIF-8/pNF can maintain excellent performance of both HER and OER. As shown in Figure 9a, the overpotential of CoP@ZIF-8/pNF is still the lowest one, with 226 mV at 10 mA cm^−2^ and 331 mV at 50 mA cm^−2^, while other catalysts are 230 and 373 mV (CoP@ZIF-8/NF), 308 and 404 mV (CoP/pNF), and 401 and 481 mV (bare NF). Likewise, compared to those three catalysts, CoP@ZIF-8/pNF has the lowest Tafel slope of 77.7 mV dec^−1^ (Figure 9b). This can be partially attributed to the adsorption effect of Zn^2+^ on OH^−^, which makes the kinetics of OER more rapid [27]. Furthermore, in Figure 9c, the R_CT_ of CoP@ZIF-8/pNF (1.24 Ω) is still much lower than CoP@ZIF-8/NF (21.29 Ω) and CoP/pNF (81.44 Ω), proving that it has higher electron transfer efficiency. With regard to the stability of this electrocatalyst, it behaves similarly to its performance in HER, with excellent performance retention shown in Figure 9d. The SEM image also proves that its structure after OER has hardly changed.

### 3.7. Overall Water Splitting Performance

**Figure 10:**
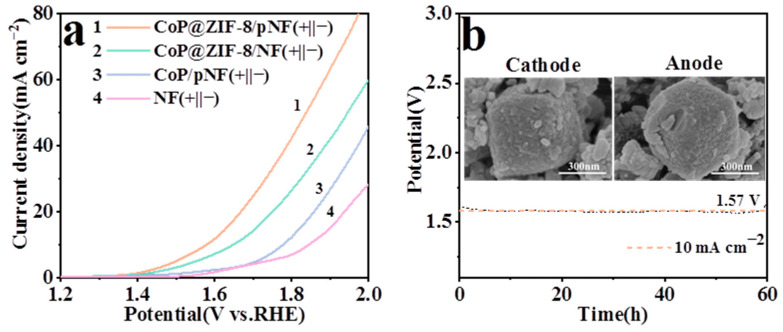
(**a**) Overall water splitting LSV curves of CoP@ZIF-8/pNF (+||−), CoP@ZIF-8/NF (+||−), CoP/pNF (+||−) and bare NF (+||−). (**b**) Stability test of water splitting at a constant current density of 10 mA cm^−2^ when using CoP@ZIF-8/pNF as an electrode, and SEM images of the anode and cathode after the stability test.

In order to measure the water splitting performance of each electrocatalyst, the as-prepared materials were assembled into two-electrode electrolytic cells as anode and cathode. As shown in Figure 10a, the CoP@ZIF-8/pNF || CoP@ZIF-8/pNF needed the lowest cell voltage of 1.57 V, reaching 10 mA cm^−2^ current density, while there was a need for 1.65 V for CoP@ZIF-8/NF || CoP@ZIF-8/NF, 1.77 V for CoP/pNF || CoP/pNF and 1.84 V for NF || NF. This indicates that the CoP@ZIF-8/pNF electrocatalyst does indeed combine the electrocatalytic properties of porous pNF and CoP@ZIF-8 with an abundant porous core–shell structure. At the same time, the stability of the ZIF-8 as the shell ensures the long-term stability of the electrocatalyst for practical water splitting applications. Therefore, CoP@ZIF-8/pNF dual functional electrocatalysts were assembled into a cell and tested for constant current electrolysis. As shown in Figure 10b, the cell could maintain the voltage of 1.57 V at a current density of 10 mA cm^−2^ for 60 h. In addition, the SEM images of the cathode and anode indicate that the catalytic active substance CoP@ZIF-8 obtained partial structural damage after long-term work, but it still maintained the stable existence of the nano-scaled core–shell structure. Compared to other works to prepare CoP and its derivatives catalysts, this method has the advantages of low energy consumption, no secondary heat treatment and clever use of the modified porous NF catalyst as a carrier. In addition, it achieves excellent performance for HER, OER and overall water splitting and the comparison with other works is detailed in Table 1.

### 3.8. Electrocatalytic Active Site and Bubble Phenomenon

**Figure 11:**
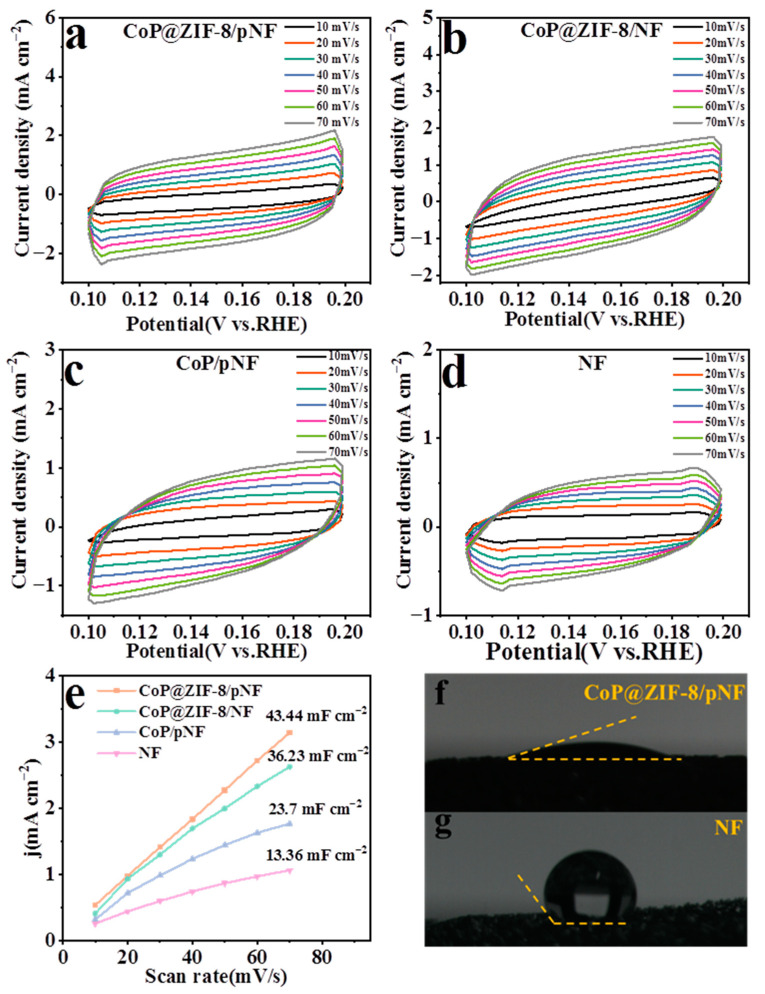
CVs at different scan rates between 0.1 and 0.2 V (vs. RHE) for CoP@ZIF-8/pNF (**a**), CoP@ZIF-8/NF (**b**), CoP/pNF (**c**) and bare NF (**d**). The C_dl_ values to evaluate the ECSA of CoP@ZIF-8/pNF, CoP@ZIF-8/NF, CoP/pNF and bare NF (**e**). The hydrophilic test of CoP@ZIF-8/pNF (**f**) and bare NF (**g**).

ECSA represents the active surface area of electrocatalysis, which is linearly proportional to the double-layer capacitance (C_dl_). Therefore, the electrocatalytic active area of different electrocatalysts can be obtained by calculating C_dl_. C_dl_ can be calculated by a CV cycles curve in a non-Faraday interval. As shown in Figure 11, CV cycles were performed on CoP@ZIF-8/pNF, CoP@ZIF-8/NF, CoP/pNF, and NF at different scan speeds in the interval 0.1–0.2 V (vs. RHE). The difference value between anode current density (J_a_) and cathode current density (J_c_) at 0.15 V (vs. RHE) can be fitted to a straight line with scan speeds, and the half value of the slope of the line is the C_dl_ of the electrocatalyst. As shown in Figure 11a–e, the C_dl_ of CoP@ZIF-8/pNF was 43.44 mF cm^−2^, which was higher than CoP@ZIF-8/NF (36.23 mF cm^−2^), CoP/pNF (23.7 mF cm^−2^) and bare NF (13.36 mF cm^−2^). The above results further indicate that the unique spatial three-level structure of CoP@ZIF-8/pNF increased the reactive area of the electrocatalyst.

Figure 11f–g shows the results of the contact angle with water of CoP@ZIF-8/pNF and NF. The contact angle can indicate the hydrophilicity of the electrocatalyst. The smaller the contact angle is, the better the hydrophilicity is, and the more favorable it is for bubbles to escape from the surface of the electrocatalyst. Compared with the NF, CoP@ZIF-8/pNF obviously has better hydrophilicity, which significantly improves its electrocatalytic performance.

## 4. Discussion

In an alkaline electrolyte, the classical two-electron step in which HER occurs consists of the following two steps:

(6) $$M+H_{2}O+e^{-}\to {M–H}_{ads}+OH^{-}$$

(7) $${M–H}_{ads}+H_{2}O+e^{-}\to H_{2}+M+OH^{-}$$

Formula (6) is the HER Volmer process, which is the speed determination step [63]. Formula (7) is the subsequent Heyrovsky process. CoP, with its isotropic crystal structure and abundant coordination unsaturated metal surface atoms, accelerates Formula (6) as a core part of the CoP@ZIF-8/pNF electrocatalyst. The pore and channel structure of ZIF-8 and the porous pNF are conducive to the rapid desorption of H_2_ and make Formula (7) proceed faster. CoP interacted with the electrolyte and transferred bubbles through abundant channels on ZIF-8.

The corresponding OER is relatively complex and involves four electron transfer steps:

(8) $$OH^{-}+M\to {M–OH}_{ads}^{-}+e^{-}$$

(9) $${M–OH}_{ads}^{-}+OH^{-}\to {M–O}_{ads}+H_{2}O+e^{-}$$

(10) $${M–O}_{ads}+OH^{-}\to {M–OOH}_{ads}+e^{-}$$

(11) $${M–OOH}_{ads}+OH^{-}\to M+H_{2}O+O_{2}+e^{-}$$

As shown in Figure 9b, the Tafel slope of CoP@ZIF-8/pNF is 77.7 mV dec^−1^, which indicates that Formula (11) is the speed determination step [64]. In addition to the vacancy structure of CoP, the abundant atomic vacancy and the attractive effect of Zn^2+^ on ZIF-8 make the oxygen-containing intermediates adsorb well [27,65]. This also speeds up the first three steps.

The results of Tafel and EIS tests on HER and OER confirm the above analysis, and the spatial three-level structure of the electrocatalyst further facilitated HER and OER processes. In addition, CoP@ZIF-8 grew in situ directly on the pNF substrate, and its excellent binding force ensured the stable progress of water splitting, while the oxidation resistance of the material is further enhanced by the use of ZIF-8 as a protective CoP shell [59].

## 5. Conclusions

In this work, we successfully completed the modification of common NF and further used the synthesized porous metal material pNF as a growth carrier for nanocatalysts. Then, the electrocatalyst CoP@ZIF-8/pNF with a spatial three-level structure was finally prepared by natural growth and low temperature phosphating. The catalyst with millimeter-sized, micron-sized and nano-scaled porous structures showed excellent catalytic performance in both HER and OER. It had overpotentials of 77 mV at 10 mA cm^−2^ and 331 mV at 50 mA cm^−2^ for HER and OER, respectively. CoP@ZIF-8/pNF || CoP@ZIF-8/pNF needs a low cell voltage of 1.57 V to reach 10 mA cm^−2^ current density and can split water continuously for more than 55 h under these work conditions. In addition, CoP@ZIF-8 maintained a stable core–shell structure, which played an important role in preventing CoP agglomeration, maintaining material stability and controlling the nanosize of the material. The internal structure of ZIF-8 was further enriched by the nanopore structure after calcination at 300 °C, which, together with the macroporous structure of the pNF, made it very easy for hydrogen and oxygen to desorb from the catalyst interior and surface. This work provides an idea for the construction of multi-stage architectural catalysts and offers an efficient catalytic bifunctional electrocatalyst for hydrogen production applications.

## Figures and Tables

**Table 1:** Comparison of the performance of different electrocatalysts.

Catalysts	Number of Heat Treatments	Maximum Temperature	HERη (mV) (10 mA cm^−2^)	OERη (mV) (10 mA cm^−2^)	Overall Water Splitting (V) (10 mA cm^−2^)	Stability (h)	Refs.
CoP@ZIF-8/pNF	1	300 °C	77	226	1.57	60	This work
Pt/C||RuO_2_	-	-	-	-	1.57	240	[18,19]
CoP-NS/C	2	300 °C	140	292	-	24	[23]
CoP/CNFs	4	900 °C	225	325	1.65	8	[29]
CoP/C	2	800 °C	140	250	1.56	24	[30]
NiCoP NWs/NF	2	300 °C	118	-	-	48	[34]
np-CoMoP	2	>1500 °C	40.8	-	-	24	[35]
CoMoP	1	350 °C	89	273	1.56	100	[38]
Co-NC@CoP-NC	2	700 °C	-	330	-	5.5	[41]
Ag-CoP	2	400 °C	97	256	1.57	12	[44]
CoP@NC	1	350 °C	75	268	1.69	9	[45]

## Data Availability

The data are not available on reasonable request from the corresponding authors.

## References

[1] Xie G., Zhang K., Guo B., Liu Q., Fang L., Gong J.R. (2013). Graphene-based materials for hydrogen generation from light-driven water splitting. Adv. Mater..

[2] Steven C., Arun M. (2012). Opportunities and challenges for a sustainable energy future. Nature.

[3] Zhao Y., Gao W., Li S., Williams G.R., Mahadi A.H., Ma D. (2019). Solar-versus Thermal-Driven Catalysis for Energy Conversion. Joule.

[4] Liang W.A., Han X., Ren L.G. (2016). NiCoFe Layered Triple Hydroxides with Porous Structures as High-Performance Electrocatalysts for Overall Water Splitting. ACS Energy Lett..

[5] Liu Y., Jiang S., Li S., Zhou L., Li Z., Li J., Shao M. (2019). Interface engineering of (Ni, Fe)S_2_@MoS_2_ heterostructures for synergetic electrochemical water splitting. Appl. Catal. B Environ..

[6] Mikaela G., Petko C., Jorge F.d.A., Tobias R., Sören D., Benjamin P., Ralph K., Holger D., Peter S. (2016). Oxygen Evolution Reaction Dynamics, Faradaic Charge Efficiency, and the Active Metal Redox States of Ni-Fe Oxide Water Splitting Electrocatalysts. J. Am. Chem. Soc..

[7] Xu Y., Feng T., Cui Z., Guo P., Wang W., Li Z. (2021). Fe_7_S_8_/FeS_2_/C as an efficient catalyst for electrocatalytic water splitting. Int. J. Hydrogen Energy.

[8] Zhao S., Wang Y., Dong J., He C.-T., Yin H., An P., Zhao K., Zhang X., Gao C., Zhang L. (2016). Ultrathin metal–organic framework nanosheets for electrocatalytic oxygen evolution. Nat. Energy.

[9] Zhou L., Sun L., Xu L., Wan C., An Y., Ye M. (2020). Recent Developments of Effective Catalysts for Hydrogen Storage Technology Using N-Ethylcarbazole. Catalysts.

[10] Ha Y., Shi L., Chen Z., Wu R. (2019). Phase-Transited Lysozyme-Driven Formation of Self-Supported Co_3_O_4_@C Nanomeshes for Overall Water Splitting. Adv. Sci..

[11] Chen T., Tan Y. (2018). Hierarchical CoNiSe_2_ nano-architecture as a high-performance electrocatalyst for water splitting. Nano Res..

[12] Li X., Hao X., Abudula A., Guan G. (2016). Nanostructured catalysts for electrochemical water splitting: Current state and prospects. J. Mater. Chem. A Mater. Energy Sustain..

[13] Hang L., Zhang T., Sun Y., Men D., Lyu X., Zhang Q., Cai W., Li Y. (2018). Ni_0.33_Co_0.67_MoS_4_ nanosheets as a bifunctional electrolytic water catalyst for overall water splitting. J. Mater. Chem. A.

[14] Chen P., Xu K., Fang Z., Tong Y., Wu J., Lu X., Peng X., Ding H., Wu C., Xie Y. (2015). Metallic Co4N Porous Nanowire Arrays Activated by Surface Oxidation as Electrocatalysts for the Oxygen Evolution Reaction. Angew. Chem..

[15] Wang Z., Xiao B., Lin Z., Shen S., Xu A., Du Z., Chen Y., Zhong W. (2021). In-situ surface decoration of RuO_2_ nanoparticles by laser ablation for improved oxygen evolution reaction activity in both acid and alkali solutions. J. Energy Chem..

[16] Zhang B., Zheng X., Voznyy O., Comin R., Bajdich M., García-Melchor M., Han L., Xu J., Liu M., Zheng L. (2016). Homogeneously dispersed multimetal oxygen-evolving catalysts. Science.

[17] Ji S., Chen W., Zhao Z., Yu X., Park H.S. (2020). Molybdenum oxynitride nanoparticles on nitrogen-doped CNT architectures for the oxygen evolution reaction. Nanoscale Adv..

[18] He X., Dong Y., Yin F., Li G., Zhao X. (2022). NiCo_2_O_4_ nanoparticles rich in oxygen vacancies: Salt-Assisted preparation and boosted water splitting. Front. Chem..

[19] Yan L., Cao L., Dai P., Gu X., Liu D., Li L., Wang Y., Zhao X. (2017). Metal-Organic Frameworks Derived Nanotube of Nickel–Cobalt Bimetal Phosphides as Highly Efficient Electrocatalysts for Overall Water Splitting. Adv. Funct. Mater..

[20] Ma Y.Y., Wu C.X., Feng X.J., Tan H.Q., Yan L.K., Liu Y., Kang Z.H., Wang E.B., Li Y.G. (2017). Highly efficient hydrogen evolution from seawater by a low-cost and stable CoMoP@C electrocatalyst superior to Pt/C. Energy Environ. Sci..

[21] Du C., Shang M., Mao J., Song W. (2017). Hierarchical MoP/Ni_2_P heterostructures on nickel foam for efficient water splitting. J. Mater. Chem. A.

[22] Dinh K.N., Liang Q., Du C.-F., Zhao J., Tok A.I.Y., Mao H., Yan Q. (2019). Nanostructured metallic transition metal carbides, nitrides, phosphides, and borides for energy storage and conversion. Nano Today.

[23] Li H., Ke F., Zhu J. (2018). MOF-Derived Ultrathin Cobalt Phosphide Nanosheets as Efficient Bifunctional Hydrogen Evolution Reaction and Oxygen Evolution Reaction Electrocatalysts. Nanomaterials.

[24] Chen N., Che S., Liu H., Ta N., Li G., Chen F., Ma G., Yang F., Li Y. (2022). In Situ Growth of Self-Supporting MOFs-Derived Ni_2_P on Hierarchical Doped Carbon for Efficient Overall Water Splitting. Catalysts.

[25] Kang Q., Li M., Shi J., Lu Q., Gao F. (2020). A Universal Strategy for Carbon-Supported Transition Metal Phosphides as High-Performance Bifunctional Electrocatalysts towards Efficient Overall Water Splitting. ACS Appl. Mater. Interfaces.

[26] Wu J., Wang D., Wan S., Liu H., Wang C., Wang X. (2020). An Efficient Cobalt Phosphide Electrocatalyst Derived from Cobalt Phosphonate Complex for All-pH Hydrogen Evolution Reaction and Overall Water Splitting in Alkaline Solution. Small.

[27] Huang Z.-F., Song J., Du Y., Xi S., Dou S., Nsanzimana J.M.V., Wang C., Xu Z.J., Wang X. (2019). Chemical and structural origin of lattice oxygen oxidation in Co–Zn oxyhydroxide oxygen evolution electrocatalysts. Nat. Energy.

[28] Li Z., Feng H., Song M., He C., Zhuang W., Tian L. (2021). Advances in CoP electrocatalysts for water splitting. Mater. Today Energy.

[29] Xie X.Q., Liu J., Gu C., Li J., Zhao Y., Liu C.S. (2022). Hierarchical structured CoP nanosheets/carbon nanofibers bifunctional eletrocatalyst for high-efficient overall water splitting. J. Energy Chem..

[30] Li X., Qian X., Xu Y., Duan F., Yu Q., Wang J., Chen L., Dan Y., Cheng X. (2020). Electrodeposited cobalt phosphides with hierarchical nanostructure on biomass carbon for bifunctional water splitting in alkaline solution. J. Alloys Compd..

[31] Oyama S.T. (2003). Novel catalysts for advanced hydroprocessing: Transition metal phosphides. J. Catal..

[32] Zhang B., Lui Y.H., Gaur A.P., Chen B., Tang X., Qi Z., Hu S. (2018). Hierarchical FeNiP@Ultrathin Carbon Nanoflakes as Alkaline Oxygen Evolution and Acidic Hydrogen Evolution Catalyst for Efficient Water Electrolysis and Organic Decomposition. ACS Appl. Mater. Interfaces.

[33] Huang J., Su Y., Zhang Y., Wu W., Wu C., Sun Y., Lu R., Zou G., Li Y., Xiong J. (2018). FeO_x_/FeP hybrid nanorods neutral hydrogen evolution electrocatalysis: Insight into interface. J. Mater. Chem. A.

[34] Liu T., Yan X., Xi P., Chen J., Qin D., Shan D., Devaramani S., Lu X. (2017). Nickel–Cobalt phosphide nanowires supported on Ni foam as a highly efficient catalyst for electrochemical hydrogen evolution reaction. Int. J. Hydrogen Energy.

[35] Tang W., Zhu S., Jiang H., Liang Y., Li Z., Wu S., Cui Z. (2022). Self-supporting nanoporous CoMoP electrocatalyst for hydrogen evolution reaction in alkaline solution. J. Coll. Interface Sci..

[36] Yang B., Du Y., Shao M., Bin D., Zhao Q., Xu Y., Liu B., Lu H. (2022). MOF-derived RuCoP nanoparticles-embedded nitrogen-doped polyhedron carbon composite for enhanced water splitting in alkaline media. J. Coll. Interface Sci..

[37] Xu H., Cao J., Shan C., Wang B., Xi P., Liu W., Tang Y. (2018). MOF-Derived Hollow CoS Decorated with CeO_x_ Nanoparticles for Boosting Oxygen Evolution Reaction Electrocatalysis. Angew. Chem..

[38] Wang X., Yang L., Xing C., Han X., Du R., He R., Guardia P., Arbiol J., Cabot A. (2022). MOF-Derived Ultrathin Cobalt Molybdenum Phosphide Nanosheets for Efficient Electrochemical Overall Water Splitting. Nanomaterials.

[39] Assfour B., Lconi S., Seifert G. (2010). Hydrogen Adsorption Sites in Zeolite Imidazolate Frameworks ZIF-8 and ZIF-11. J. Phys. Chem. C Nanomater. Interfaces.

[40] Park K.S., Ni Z., Côté A.P., Choi J.Y., Huang R., Uribe-Romo F.J., Chae H.K., O’Keeffe M., Yaghi O.M. (2006). Exceptional chemical and thermal stability of zeolitic imidazolate frameworks. Proc. Natl. Acad. Sci. USA.

[41] Li X., Jiang Q., Dou S., Deng L., Huo J., Wang S. (2016). ZIF-67-derived Co-NC@CoP-NC nanopolyhedra as an efficient bifunctional oxygen electrocatalyst. J. Mater. Chem. A Mater. Energy Sustain..

[42] Xiao L., Zheng S., Yang K., Duan J., Jiang J. (2021). The construction of CoP nanoparticles coated with carbon layers derived from core-shell bimetallic MOF for electrochemical detection of dopamine. Microchem. J..

[43] Wang J., Chen C., Cai N., Wang M., Li H., Yu F. (2021). High topological tri-metal phosphide of CoP@FeNiP toward enhanced activities in oxygen evolution reaction. Nanoscale.

[44] Tong X., Liao W., Fu Y., Qian M., Dai H., Mei L., Zhai Y., Chen T., Yang L., Yang Q. (2022). Ag-doped CoP Hollow Nanoboxes as Efficient Water Splitting Electrocatalysts and Antibacterial Materials. ChemistrySelect.

[45] Li Z., Sui J., Zhang Q., Yu J., Yu L., Dong L. (2020). CoP@NC electrocatalyst promotes hydrogen and oxygen productions for overall water splitting in alkaline media. Int. J. Hydrogen Energy.

[46] Jomekian A., Bazooyar B., Esmaeilzadeh J., Behbahani R.M. (2020). Highly CO_2_ selective chitosan/g-C_3_N_4_/ZIF-8 membrane on polyethersulfone microporous substrate. Sep. Purif. Technol..

[47] Wang Q., Ji Y., Shi J., Wang L. (2020). NIR-driven Water Splitting H2 Production Nano-platform for H_2_ Mediated Cascade Amplifying Synergetic Cancer Therapy. ACS Appl. Mater. Interfaces.

[48] Zhang J., Li C.M. (2012). Nanoporous metals: Fabrication strategies and advanced electrochemical applications in catalysis, sensing and energy systems. Chem. Soc. Rev..

[49] Nam D.H., Kim R.H., Lee C.L., Kwon H.S. (2012). Highly Reversible Sn. J. Electrochem. Soc..

[50] Zhang W., Ding C., Wang A., Zeng Y. (2015). 3-D Network Pore Structures in Copper Foams by Electrodeposition and Hydrogen Bubble Templating Mechanism. J. Electrochem. Soc..

[51] Plowman B.J., Jones L.A., Bhargava S.K. (2015). Building with bubbles: The formation of high surface area honeycomb-like films via hydrogen bubble templated electrodeposition. Chem. Commun..

[52] Zhang H., Ye Y., Shen R., Ru C., Hu Y. (2013). Effect of Bubble Behavior on the Morphology of Foamed Porous Copper Prepared via Electrodeposition. J. Electrochem. Soc..

[53] Yu X., Wang M., Wang Z., Gong X., Guo Z. (2016). The structure evolution mechanism of electrodeposited porous Ni films on NH_4_Cl concentration. Appl. Surf. Sci..

[54] Yu X., Yang J., Sui Z., Wang M. (2021). Effects of ultrasonic field on structure evolution of Ni film electrodeposited by bubble template method for hydrogen evolution electrocatalysis. J. Solid State Electrochem..

[55] Dong Y., Ji S., Wang H., Linkov V., Wang R. (2022). In-site hydrogen bubble template method to prepare Ni coated metal meshes as effective bi-functional electrodes for water splitting. Dalton Trans..

[56] Zhang W., Liu Y., Zhou H., Li J., Yao S., Wang H. (2019). A high-performance electrocatalyst of CoMoP@NF nanosheet arrays for hydrogen evolution in alkaline solution. J. Mater. Sci..

[57] Zhu Z., Ma J., Xu L., Xu L., Li H., Li H. (2012). Facile Synthesis of Co–B Amorphous Alloy in Uniform Spherical Nanoparticles with Enhanced Catalytic Properties. ACS Catal..

[58] Bai X.-J., Zhai X., Zhang L.-Y., Fu Y., Qi W. (2021). Site-directed reduction engineering within bimetal-organic frameworks for efficient size-selective catalysis. Matter.

[59] Yun Y., Fang Y., Fu W., Du W., Zhu Y., Sheng H., Astruc D., Zhu M. (2022). Exploiting the Fracture in Metal-Organic Frameworks: A General Strategy for Bifunctional Atom-Precise Nanocluster/ZIF-8(300 °C) Composites. Small.

[60] Pan Y., Sun K., Liu S., Cao X., Wu K., Cheong W.C., Chen Z., Wang Y., Li Y., Liu Y. (2018). Core-Shell ZIF-8@ZIF-67-Derived CoP Nanoparticle-Embedded N-Doped Carbon Nanotube Hollow Polyhedron for Efficient Overall Water Splitting. J. Am. Chem. Soc..

[61] Lin Y., Liu M., Pan Y., Zhang J. (2017). Porous Co–Mo phosphide nanotubes: An efficient electrocatalyst for hydrogen evolution. J. Mater. Sci..

[62] Jin J., Yin J., Liu H., Huang B., Hu Y., Zhang H., Sun M., Peng Y., Xi P., Yan C.H. (2021). Atomic Sulfur Filling Oxygen Vacancies Optimizes H Absorption and Boosts the Hydrogen Evolution Reaction in Alkaline Media. Angew. Chem..

[63] Bai N., Li Q., Mao D., Li D., Dong H. (2016). One-Step Electrodeposition of Co/CoP Film on Ni Foam for Efficient Hydrogen Evolution in Alkaline Solution. ACS Appl. Mater. Interfaces.

[64] Zhang W., Lu Y., Wang H., Yao S. (2021). Self-Assembled MoO_x_@Co_2_P_4_O_12_ as an Ideal Bifunctional Catalyst for Overall Water Splitting. J. Electrochem. Soc..

[65] Xu J., Li J., Xiong D., Zhang B., Liu Y., Wu K.H., Amorim I., Li W., Liu L. (2018). Trends in activity for the oxygen evolution reaction on transition metal (M = Fe, Co, Ni) phosphide pre-catalysts. Chem. Sci..

